# Primary chondroma of posterior mediastinum with Horner’s syndrome: a case report

**DOI:** 10.1186/s12957-018-1511-6

**Published:** 2018-10-17

**Authors:** Xiaoqian Xu, Xin Li, Fan Ren, Ming Dong, Minghui Liu, Jun Chen

**Affiliations:** Department of Lung Cancer Surgery, Tianjin Key Laboratory of Lung Cancer Metastasis and Tumor Microenvironment, Tianjin Lung Cancer Institute, Tianjin Medical University General Hospital, Anshan Road No.154, Heping District, Tianjin, 300052 China

**Keywords:** Primary mediastinal mass, Chondroma, Horner’s syndrome

## Abstract

**Background:**

Chondroma is a slowly growing, benign cartilaginous tumor which predominantly occurs in long bones of the hands and feet. Primary mediastinal chondroma is rare, especially with Horner’s syndrome.

**Case presentation:**

We reported the case of a 31-year-old woman with a posterior mediastinum mass associated with Horner’s syndrome. After complete dissection of the mass, a pathological diagnosis of the primary mediastinal chondroma was rendered. The patient has shown no local recurrence or distal disease in a 3.5-year follow-up period.

**Conclusions:**

The preoperative diagnosis of chondroma should combine various examinations for comprehensive evaluation. Complete surgical resection should be the first choice of the treatment due to the risk of malignancy.

## Background

Chondroma is a slowly growing, benign cartilaginous tumor which predominantly occurs in long bones of the hands and feet. Primary mediastinal chondroma is rare. Besides, Horner’s syndrome which is characterized by ipsilateral ptosis, miosis, and anhydrosis has been seldom described in such tumors before. Here we report the case of a primary chondroma located in posterior mediastinum which is associated with Horner’s syndrome.

## Case presentation

**Fig. 1:**
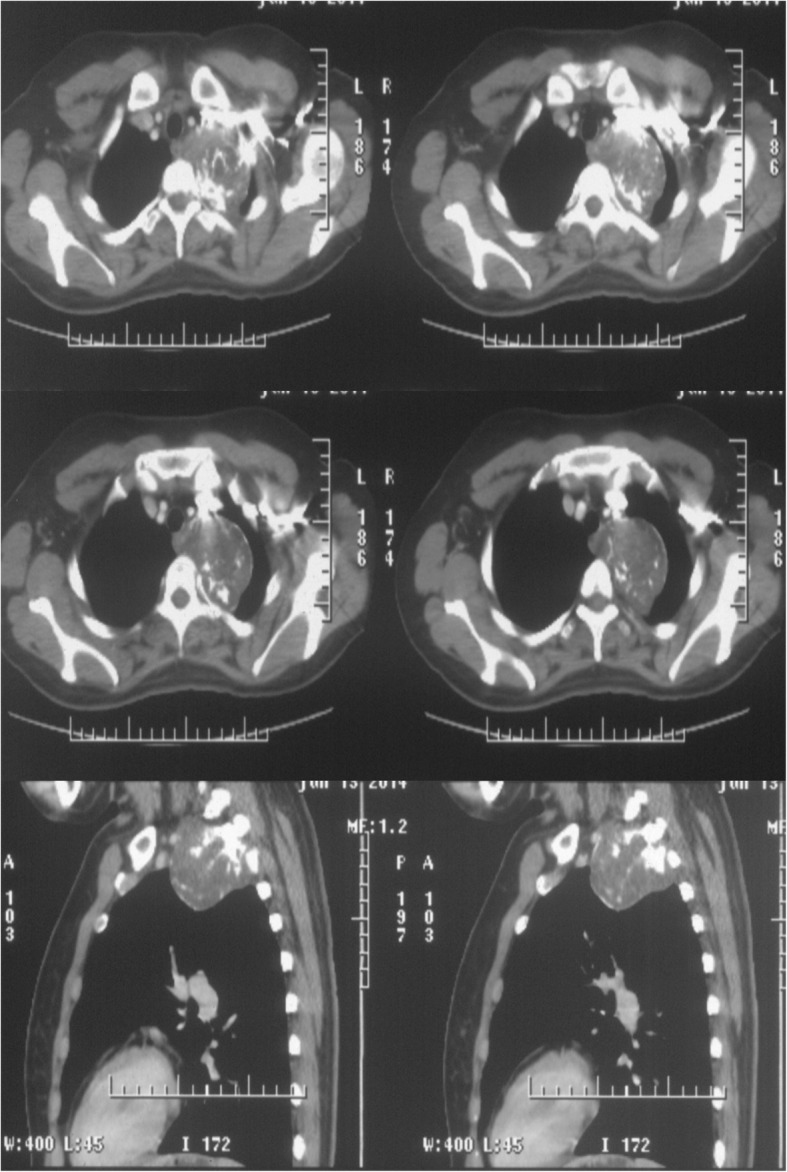
The contrast-enhanced CT scans of the chest. There was a round, soft tissue density mass in the posterior superior mediastinum. The mass with high-density shadow was related to the left second rib in which soup bubbly appearance and expansion can be found

A 31-year-old woman was admitted to our hospital with the chief complaints of anhydrosis on the left upper limb, ipsilateral face, and miosis. These symptoms developed without apparent causes and in the absence of other problems, such as ptosis, enophthalmus, fever, chest pain, breathlessness, cough, expectoration, nausea, or vomiting. Contrast-enhanced computed tomography of the chest revealed a 6.1 × 5.6 × 5.5-cm, well-circumscribed soft tissue mass in the left posterior mediastinum (Fig. 1). In addition, adjacent intervertebral foramen of thoracic vertebra became larger, and bone destruction of the left second rib can also be seen. Particularly, there was no enlarged lymph node within the mediastinum.

**Fig. 2:**
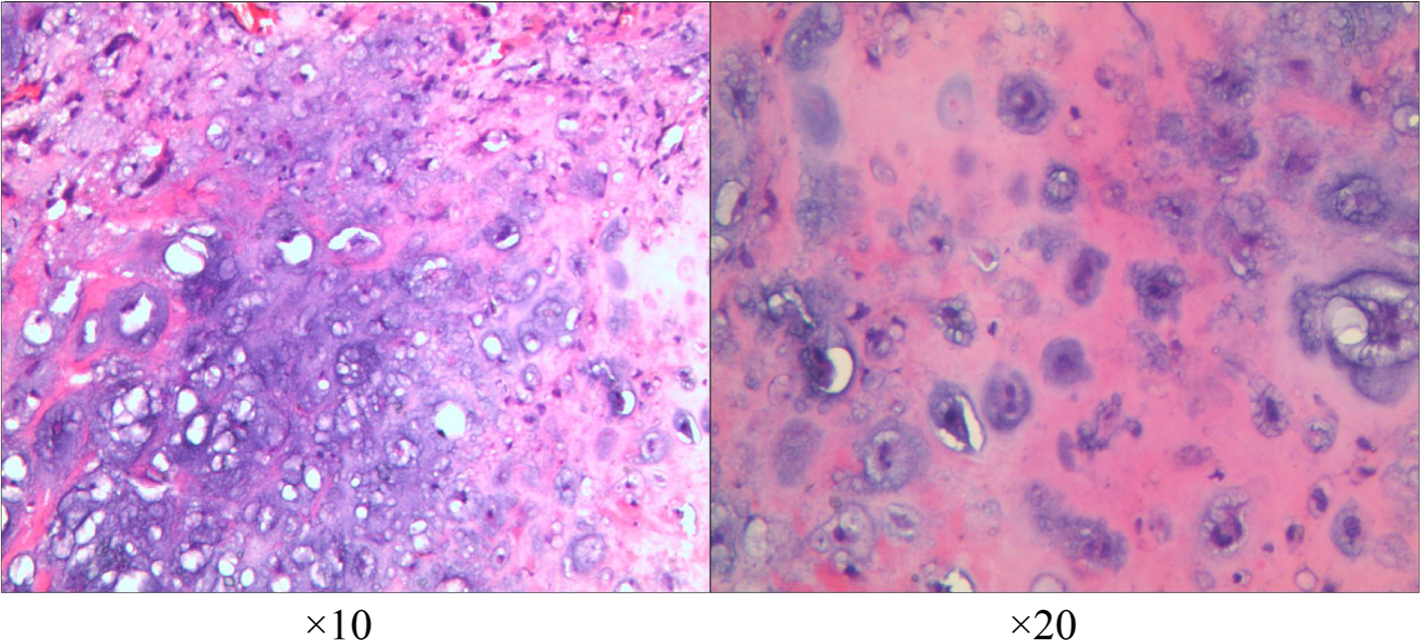
Pathological characteristics. The pathologic examination showed the chondroma with dense chondrocytes and active growth by H&E staining

A left posterolateral thoracotomy through the fifth intercostal space was performed. During the surgery, it was found that the mass which was approximately 6 × 5 × 5 cm in size arose from the cortex of the second rib and was hard in consistency. Ossification could also be seen within the tumor. We resected the tumor completely and removed partial sclerotin on the surface of the second rib. Histopathologic examination confirmed the diagnosis of chondroma (Fig. 2). Her postoperative course was uneventful. She was discharged without any complication in 12 days after surgery. Follow-up high-resolution computed tomography scans have not detected recurrence 42 months after surgery.

## Discussion

Chondroma is a benign bone tumor that originates from the cartilage tissue. It predominantly occurs in long bones of the hands and feet. It is rare to be found in thoracic cavity, including the lungs, bronchi, and mediastinum [1–3]. Only a few cases of chondroma in the mediastinum have been reported [1–3]. Similar to this case, Zhao et al. reported a giant chondroma originating from the fourth rib on the left [3]. It can occur in any age group, and there is no obvious gender difference. Chondroma in the lungs or mediastinum is usually asymptomatic and discovered incidentally on routine chest radiography. The computed tomography scan of the chest mostly displays a solitary round or oval-shaped nodule or mass with clear borders and even dense in which calcified speckles can be seen. The patient may have symptoms that include chest pain, irritating cough, or neurological abnormalities due to compression or direct invasion of surrounding mediastinal structures. Macroscopically, the tumor is gray-white translucent, and the lobes can be seen in the section. Microscopically, the tumor is composed of well-differentiated cartilage tissue and surrounded by cartilage matrix. In this case, the contrast-enhanced computed tomography revealed a round and soft tissue density mass in posterior superior mediastinum in which nodular and lined high-density shadow can be seen. And the tumor was related to the left second rib in which soup bubbly appearance and expansion can be found. The patient had Horner’s syndrome including symptoms like left ptosis, miosis, enophthalmos, and facial anhydrosis as a result of the compression of the cervical sympathetic nerve.

## Conclusion

The preoperative diagnosis of chondroma should combine various examinations for comprehensive evaluation. At the same time, we should differentiate it with lung hamartoma, tuberculosis, and other diseases. Complete surgical resection should be the first choice of the treatment due to the risk of malignancy [4]. We usually choose partial lung resection or lobectomy for pulmonary chondroma and tumor resection for mediastinal chondroma [5]. Surgical treatment is effective for chondroma, and the tumor is rare to relapse and metastasize.

## Abbreviation

CT: Computed tomography
